# Current trends and prospects in quinoa research: An approach for strategic knowledge areas

**DOI:** 10.1002/fsn3.3891

**Published:** 2023-12-21

**Authors:** Diego Hernando Flórez‐Martínez, Jader Rodríguez‐Cortina, Luis Fernando Chavez‐Oliveros, Germán Andrés Aguilera‐Arango, Alexis Morales‐Castañeda

**Affiliations:** ^1^ Corporación Colombiana de Investigación Agropecuaria (AGROSAVIA)—Sede Central Mosquera Colombia; ^2^ Corporación Colombiana de Investigación Agropecuaria (AGROSAVIA)—Centro de Investigación Tibaitatá Mosquera Colombia; ^3^ Centro de Investigación de la Caña de Azúcar de Colombia (CENICAÑA) Florida Valle del Cauca Colombia; ^4^ Corporación Colombiana de Investigación Agropecuaria (AGROSAVIA)—Centro de Investigación Palmira Palmira Valle del Cauca Colombia; ^5^ Corporación Colombiana de Investigación Agropecuaria (AGROSAVIA)—Sede Central Mosquera Colombia

**Keywords:** andean grains, functional foods, plant‐based proteins, productive chains, quinoa, scientometrics, trend analyses

## Abstract

Currently, the demand for healthy consumption and the use of alternatives to dairy proteins for the development of foods with good nutritional value are growing. Quinoa has received much attention because it contains a high content of proteins, essential amino acids, essential fatty acids, minerals, vitamins, dietary fibers, and bioactive compounds. Nevertheless, this content and the bioavailability of specific compounds of interest are related to the genotype, the agri‐environmental conditions, and management practices where quinoa is grown and postharvest management. This article aimed to analyze the research trends for three knowledge areas: quinoa plant breeding for nutraceutical properties, plant–soil relations focused on abiotic stresses, and postharvest and value‐added transformation activities. To this end, a specific methodological design based on bibliometrics and scientometrics methods was used. Through these analyses based on publications' keywords, titles, abstracts, and conclusions sections, for each knowledge area, the key research trends (scope and main topics), the classification of trends based on their development and relevance degree, and the core of knowledge were established. The trends comprise the current state of research. Finally, analyzing the conclusions, recommendations, and future research sections of key publications, a strong correlation among plant breeding research to obtain varieties with tolerance to biotic and abiotic stresses, nutritional and functional compounds of interest for food safety, and the development of products with higher added value established interest in further research on the potential bioactivity of quinoa and the verification of health benefits to humans.

## Graphical abstract

Functional food research has diversified product, process, and packing design to enhance nutritional, organoleptic, and healthy characteristics. Quinoa (*Chenopodium quinoa* Willd) has been focused on promoting nutritional, health, cosmetic, and animal feed uses based on bioactive compound contents. The technological development of quinoa crops includes genetic enhancement methods, soil–plant relations improvement, and postharvesting and value‐added processes. Quinoa can be used as a valuable ingredient in the formulation of foods with value added and improved nutritional characteristics with higher contents of all essential amino acids, especially lysine. Quinoa crops contribute to bioeconomy trends in agriculture and food sector in Latin‐American countries.

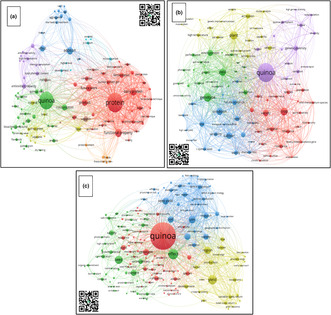

## 1 INTRODUCTION

The Andean region in South America comprises the territories of Colombia, Ecuador, Perú, and Bolivia, which have been recognized for their biodiversity and plant richness. These natural regions embrace a germplasm collection of fruits, vegetables, tubers, roots, cereals, and pseudocereals known as “Andean crops.” These crops are grown mainly in marginal areas (in contrast to extensive crops in modern agriculture) and are characterized by their nutritional content and potential application of bioactive compounds (Salazar et al., 2021).

Andean crops have become a major trend in food safety due to their bioactive compound content, not only for scientific research but also in food markets for the consumption of products with high‐quality requirements of inputs, intermediate products, and final products (Hermann, 2009). Furthermore, Andean crops can contribute to the health, cosmetic, and pharmaceutical industries mainly for starch and flour derivatives (Wongsagonsup et al., 2021). One of the most recognized Andean crops worldwide is quinoa, a pseudocereal used as a functional food that is associated with numerous health benefits (López‐Cervantes et al., 2021).

Quinoa (*Chenopodium quinoa* Willd) is a pseudocereal belonging to the Amaranthaceae family and is important worldwide due to its nutritional value since its seeds contain high levels of proteins, lipids, vitamins, minerals, and essential amino acids such as lysine, methionine, and cysteine; thus, its cultivation is promoted in countries with food security problems (Bazile et al., 2021; Gomez‐Pando et al., 2019; Zurita‐Silva et al., 2014). In addition to its nutritional properties, quinoa has aroused interest in studies due to its wide adaptation to different agri‐environmental conditions, thanks to its genetic diversity, which is observed in the expression of morphological, phenological, and metabolic characteristics (Gutiérrez & Portugal, 2022). According to Subedi et al. (2021), Quinoa (*Chenopodium quinoa*) is an allotetraploid species with a subgenome composition of AABB (2*n* = 4*x* = 36) from hybridization between diploid species of the genus *Chenopodium* with AA and BB genome accompanied by a chromosome duplication event. This hybridization event is estimated to have occurred 3.3–6.3 million years ago in North America, where in addition to *C*. *quinoa*, *C*. *berlandieri* Moq., and *C*. *hircinum* Schrad, they also share the composition of the allotetraploid AABB subgenome. These three species together make up the allotetraploid goosefoot complex (ATGC) that underwent long‐range dispersal, leading to the emergence and domestication of quinoa in South América, in the Lake Titicaca basin in Peru and Bolivia. Subsequently and in accordance with Ticona et al. (2022), the natural geographic distribution of the species extends from 5° north latitude in southern Colombia (García‐Parra, Stechauner‐Rohringer, et al., 2020; García‐Parra, Zurita‐Silva, et al., 2020; Manjarres‐Hernández & Morillo‐Coronado, 2022) to 43° south latitude in the Tenth Region of Chile (Fuentes et al., 2012) and Argentina (Vidueiros et al., 2015), while the altitudinal distribution ranges between 0 and 4000 m.a.s.l. Thus, quinoa crops have a high adaptability to different agroclimatic conditions, making them one of the crops with the best productive potential to be adopted by small producers with a great contribution to food safety (Bazile et al., 2015; Camaggio, 2014; Garcia et al., 2018).

As reported by Tapia (2000) cited by Schmidt et al. (2021), five different ecotypes have been described for quinoa growth: (1) valley ecotype; (2) altiplanic ecotype; (3) salares ecotype; (4) sea level ecotype; and (5) subtropical or Yungas ecotype. The valley ecotype is found between 2000 and 4000 m.a.s.l. in the Andean valleys, represented by the Amarilla, Maranganí, Blanca de Junín, Dulce de Lazo, Dulce de Quitopamba, Nariño, and Rosada de Juní varieties. The Altiplanic ecotype is established between 3600 and 3800 m.a.s.l., near Lake Titicaca, where the Blanca de Julí, Cheweca, Kancolla, Tahuaco, and Witulla varieties predominate, and the Salares ecotype is found between 3700 and 3800 m.a.s.l, in the Bolivian Uyuni salt flat, where the royal quinoa variety is the most representative. Finally, the sea level and subtropical ecotypes are the least studied to date, although they are gaining interest in being productive in areas where quinoa is not traditionally grown (Tapia, 2000). This remarkable adaptability reflects the great genetic diversity and exceptional adaptability of the species (Jacobsen et al., 2003).

The food market trend for healthy, environmentally friendly, and safe products has increased. This can be attributed mostly to the rise in consumer awareness (Khan et al., 2013). Additionally, there are some trends in changing food markets, such as Angeli et al. (2020), that can be related to quinoa market opportunities:

- Free‐from foods: dairy‐ and gluten‐free foods and plant‐based diets to reduce meat consumption.
- Natural foods: the increasing demand for natural foods.
- Functional foods: value‐added products that provide additional benefits to one's health.
- Energy‐boosting foods: providing long‐lasting energy in the form of calories.

Quinoa production started to steadily increase in 2013, which was the international year of quinoa, and since then, the production and consumption of quinoa have increased exponentially (Wang, Wang, et al., 2020; Wang, Zhang, et al., 2020). In 2021, according to the FAOStat portal, quinoa crops worldwide covered 192,000 ha with a production of 147,000 tons. Quinoa is emerging as a quality source of protein, fiber, minerals, and bioactive compounds with high potential in the development of gluten‐free and naturally nutrient‐enriched novel food products. Meanwhile, the market for quinoa consumption is aligned with the tendency of healthy diets by consumers.

Quinoa's importance and relevance in Colombia have been the focus of the national research agenda for the agricultural sector. The quinoa research agenda for Colombia comprises 104 scientific requirements, 14% associated with postharvest and transformation issues, 10% with plant breeding for important traits, and 10% with soil and water management. Furthermore, quinoa crop areas have increased from 145 ha in 2009 to 700 ha in 2019, with an increase in production from 253.84 tons to 1390.00 tons and an average yield of 2.17 tons per ha (MADR & UPRA, 2021). Among the research demands for the quinoa chain in Colombia, the following stand out: plant breeding to obtain improved varieties, the integral management of the productive system, georeferentiation to identify potential regions for the cultivation of quinoa, recognition and integrated management of pests and diseases, sustainable soil and water management, and fertilization and technology transfer to add value to quinoa (MADR, 2021).

The main goal of this research was to identify and characterize the current research trends for quinoa crops on sowing material and genetic enhancement, soil–plant relations, and postharvesting and added value reported in the scientific literature. Furthermore, based on key publications related to these three knowledge areas, future directions of research involving the three areas were proposed.

## 2 MATERIALS AND METHODS

**FIGURE 1:**
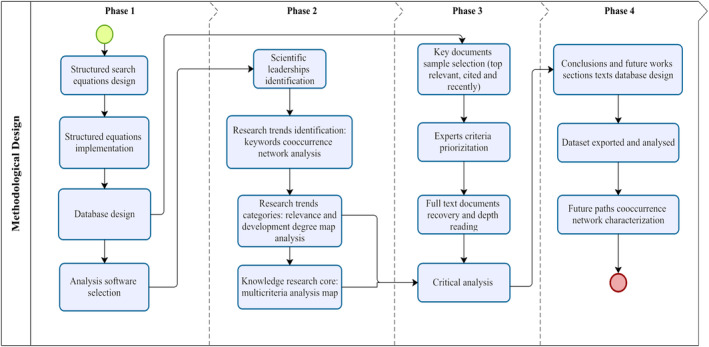
Methodological Design. *Source*: Based on Flórez‐Martínez (2022) and Flórez‐Martínez et al. (2021).

The methodological design proposed for this research consists of four sequential phases, each with specific stages (Figure 1). Furthermore, the design integrates conceptual and practical approaches of scientific and technological surveillance for trend identification (Escorsa & Maspons, 2001; Porter, 2005; Wang et al., 2021); scientometric analysis for measuring, classifying, and representing science thematic clusters and topics related to trends (Leydesdorff & Milojević, 2015); and futures analysis for short‐, middle‐, and long‐term milestone design (Popper, 2008). In further sections, each phase is described.

### 2.1 Phase 1—Data search and recovery information strategy

This phase comprises four stages based on the principles of structural equation design and data recovery from specialized scientific and technological databases. Its main goal is the consolidation of a scientific and technological information dataset for each knowledge area.

#### 2.1.1 Stage 1.1—Structured equations

Three structured equations were designed for three knowledge areas on quinoa crop research. Each one embraces three components: (i) study object, key terms related to the research interest topic (quinoa); (ii) job object, key terms related to each knowledge area such as research topics, tools, methods, or methodologies that affect the study object (sowing material and genetic improvement; soil–plant relations; and postharvest and value addition activities); and (iii) search restrictions, related to time lapse, document type, research area, or geographic area.

#### 2.1.2 Stage 1.2—Structured equations implementation

The three equations were assessed on three scientific databases, Scopus® indexing platform, Elsevier®: Web of Science®, and SciELO® from Clarivate Analytics®. The data recovered include records from January 2010 to May 2022 limited to the agriculture and biological sciences subject area and research articles, review articles, and conference proceeding documents. Datasets were downloaded both with *.csv and *.bib formats for synonymizing and normalizing data activities.

#### 2.1.3 Stage 1.3—Database design

Datasets were exported into Excel® software for duplicated registry removal and main database conformation (combining SciELO®, Scopus®, and WoS® datasets). The main database was transformed into a *.csv file and imported into specialized software.

#### 2.1.4 Stage 1.4—Software selection

The software packages selected for database analysis were Bibliometrix® 4.0.0, focusing on bibliometric analysis for top authors, institutions, journals, countries, and topics (Aria & Cuccurullo, 2017), and VOSviewer® for scientific landscape construction (Van Eck & Waltman, 2010).

### 2.2 Phase 2—Scientific and technological trend construction

In this phase, software tools were used to perform specific bibliometric and scientometric analyses for research trend identification.

#### 2.2.1 Stage 2.1—Scientific leadership identification

In this stage, data were identified and categorized based on the number of publications and citations for each thematic area: (i) top researchers for future collaboration and peer review processes; (ii) top institutions for future alliances on project formulation, technology transfer, and capacity enhancement; (iii) top countries for further benchmarking exercises or technical and scientific exploration missions; and (iv) top journals for further research result dissemination and consultation.

#### 2.2.2 Stage 2.2—Research trend identification

In this stage, co‐occurrence network analysis was conducted using VOSviewer® according to Kostoff and Schaller (2001) and Rodríguez‐Salvador et al. (2017) to identify the relationships among thematic clusters and key topics for both of the actual state of research (based on document keywords). The research trend description focuses on the creation of a narrative of each thematic cluster based on the number of topics (nodes), the number of links (connections), and the relationship cohesion (link strength). Each thematic cluster was assigned a name that embraces the main related topics.

#### 2.2.3 Stage 2.3—Research trend categories

In this stage, thematic map analysis was conducted using Bibliometrix® for thematic cluster categorization in a four‐quadrant framework. Quadrant 1 (upper right) integrates key motor themes (“mainstream”) with high scientific development (density) and high importance in the research field (centrality). Quadrant 2 (upper left) integrates highly developed isolated/niche themes (“ivory towers”) with high scientific development but low importance in the research field. Quadrant 3 (down left) integrates emerging or declining themes (chaos/unstructured) with low importance and development, otherwise known as future motor/isolated themes. Quadrant 4 (right) integrates basic and transversal themes (“bandwagon”) with high importance and low‐to‐middle development, comprising past themes, promising times, or inter‐/multidisciplinary themes (Garcia‐Garcia & Rodríguez, 2018). The description focuses on the creation of a narrative of each categorization quadrant based on the research advances of the clusters of quinoa research.

#### 2.2.4 Stage 2.4—Knowledge research core

In this stage, a factorial analysis (reducing dimensionality techniques) based on multiple correspondence analysis was performed on Bibliometrix® to identify the key subfields for each thematic area of quinoa research. These subfields comprise the core knowledge areas on which the related topics (keywords) could be highly related (short distance) or weakly related (long distance; Cuccurullo et al., 2016). The description focuses on the creation of a narrative for each knowledge core as the main subfields (pillars) for each knowledge area.

### 2.3 Phase 3—Critical analysis

This phase comprises four stages. First, from the unified database for each thematic area, a representative sample of documents was selected based on scientometric criteria (top relevant papers based on semantic coincidence between structural equation key terms and title, abstract, and keyword frequency and intensity co‐occurrence of these terms; top cited documents; and most recent documents (2021 and 2022 publication years)). Second, the documents were analyzed by experts in the field and prioritized by title and abstract reading. Third, selected documents were recovered in full text for depth reading. Fourth, based on the contribution of the selected documents, the descriptive analysis was enhanced, including critical statements that combine paper evidence (explicit knowledge) and expert experience (tacit knowledge).

### 2.4 Phase 4—Future analysis

This phase comprises the combination of tech mining principles (Porter, 2007) and scientometrics in three stages. First, a database was designed and structured by extracting conclusions and future work sections from Section 3.1 documents. Second, the dataset for VOSviewer® was exported from the database and analyzed using cooccurrence analysis for text. Third, the future work co‐occurrence network was characterized by experts from both a descriptive and critical perspective.

## 3 RESULTS AND DISCUSSION

In the following sections, the research results and discussion are presented according to the proposed methodological design.

### 3.1 Phase 1—Results

Table 1 comprises the key scientometric indicators for scientific leadership in each thematic area. Based on the proposed structural equations, the top five leaders, institutions, journals, and countries were identified. These top references contribute to monitoring potential alliances at the geopolitical level (countries interactions), mainly for regional and transnational breeding programs for quinoa biodiversity protection and origin center recognition; sharing of genetic resource advances mainly in abiotic and biotic factors resistance and the conservation of materials through in situ and ex‐situ gene and seed banks. China, the United States, and Italy could be excellent allies for countries like Perú, Colombia, Ecuador, and Chile. Regarding institutional‐level knowledge, universities and research centers that lead current development through soil–plant relationships can promote technology tranfer programs design and technical missions both for capacities strengthening and researcher exchange (visiting/stays/postdoctoral), mainly with the University of Copenhagen (Denmark), Washington State University (United States), and University of Agriculture (Pakistan). Finally, constructing longtime research alliances has its basis in researcher–researcher interactions, mainly started on conference meetings, specialized social networks (ResearchGate, Academia, Google Scholar, and LinkedIn), and general social networks (X/Twitter). Outstanding researchers for postharvest and value‐adding thematic are Jacobse, S.E (University of Copenhagen), Arendt, E.K (Ireland University College Cork), and Murphy, K.M (Washington State University). The interactions among researchers, institutions, and countries are presented in Data S1.

**TABLE 1:** Research thematic areas on quinoa, top researchers, institutions, countries, articles, and journals 2010–2022.

Research strategic focus	Search strategy (structural equation)	Leader researchers	Leader institutions	Leader countries	Top diffusion journals
Sowing material and plant breeding: 1.567 documents retrieved (Documents H‐index = 70)	TITLE‐ABS‐KEY ((“*Chenopodium quinoa*” OR “quinua” OR “quinoa” OR “quingua” OR “triguillo” OR “trigo inca” OR “arrocillo” OR “arroz del peru” OR “Kinoa” OR “lagrima de sol”) AND (“gene pool” OR “morphological character*“OR “cultivar*“OR “plant breeding” OR “germplasm*“OR “genetic resource*“OR “seed*“OR “variet*“OR “genotypic character*“OR “phenotypic character*“OR “genetic improvement” OR “genotyp*“OR “phenotyp*“OR “germplasm collection*“OR “seed collection” OR “clon*“OR “native genetic resources” OR “plant variet*“OR “germplasm bank*“OR “genetic expression” OR “genetic control” OR “genetic variability” OR “varietal description” OR “seed production” OR “gen*“OR “genetic” OR “geno*“OR “selective breeding” OR “genetic map” OR “landrace*“OR “ecotyp*“OR “propagation material” OR “accession*“OR “hybrid*“OR “certified seed” OR “vegetal material” OR “phenolog*“OR “plant breeding”)) AND PUBYEAR >2009 AND (LIMIT‐TO (DOCTYPE, “ar”) OR LIMIT‐TO (DOCTYPE, “re”) OR LIMIT‐TO (DOCTYPE, “cp”)) AND (LIMIT‐TO (SUBJAREA, “AGRI”) OR LIMIT‐TO (SUBJAREA, “BIOC”))	Jacobsen S.E.; Københavns Universitet, Department of Plant and Environmental Sciences, Copenhagen, Denmark [52–2312]	University of Copenhagen, Denmark [57–2351]	China [238–2697]	Plants [51–322]
		Shabala, Sergey; University of Tasmania, Hobart, Australia [23–902]	Consejo Nacional de Investigaciones Científicas y Técnicas, Argentina [49–745]	United States [151–2111]	Food chemistry [49–2940]
		Murphy, K.M.; Washington State University Pullman, Sustainable Seed Systems Lab, Pullman, United States [21–565]	University of Buenos Aires, Argentina [39–510]	Italy [101–2946]	Plant disease [34–95]
		Iqbal, Shahid; Muhammad Nawaz Shareef University of Agriculture, Multan, Pakistan [14–194]	National Agrarian University La Molina, Peru [38–961]	Spain [100–1545]	Frontiers in Plant Science [32–689]
		Basra, S.; University of Agriculture, Faisalabad, Faisalabad, Pakistan [13–91]	Washington State University Pullman, United States [31–587]	Argentina [86–1595]	Journal of Cereal Science [31–670]
Plant–soil relations: 421 documents retrieved (Documents H‐index = 40)	TITLE‐ABS‐KEY ((“*Chenopodium quinoa*” OR “quinua” OR “quinoa” OR “quingua” OR “triguillo” OR “trigo inca” OR “arrocillo” OR “arroz del peru” OR “Kinoa” OR “lagrima de sol”) AND (“carbon dioxide” OR “fertiliz*“OR “grain cropping” OR “crop rotation*“OR “greenhouse gas emissions” OR “GHG” OR “methane” OR “nitrous oxide” OR “soil quality index” OR “sustainable yield” OR “no‐tillage” OR “global warming potential” OR “no‐till” OR “carbon sequestration” OR “soil management” OR “abiotic stress*“OR “environmental stress*“OR “sustainable production” OR “sustainability production” OR “climate change” OR “climate resilience” OR “climate variability” OR “ecological adaptation” OR “ecophysiolog*“OR “ecosystem service*“OR “circular economy” OR “organic production” OR “food security”)) AND PUBYEAR >2009 AND (LIMIT‐TO (DOCTYPE, “ar”) OR LIMIT‐TO (DOCTYPE, “re”) OR LIMIT‐TO (DOCTYPE, “cp”))	Jacobsen S.E.; Københavns Universitet, Department of Plant and Environmental Sciences, Copenhagen, Denmark [29–1413]	University of Copenhagen, Denmark [50–1666]	China [253–755]	Plants [41–188]
		Choukr‐Allah, Redouane Mohammed VI Polytechnic University, Ben Guerir, Morocco [11–133]	Washington State University, United States [39–699]	Italy [147–1709]	Journal of Agronomy and Crop Science [41–526]
		Murphy, Kevin M. Washington State University Pullman, Pullman, United States [10–395]	University of Agriculture, Pakistan [35–214]	United States [116–1304]	Agronomy [41–94]
		Hirich, Abdelaziz Mohammed VI Polytechnic University, Ben Guerir, Morocco [9–100]	University of Florence, Italy [27–131]	Pakistan [100–375]	Frontiers in Plant Science [41–431]
		Bazile, Didier CIRAD, Paris, France [8–457]	University of Tasmania, Australia [25–361]	Morocco [95–94]	Plant Physiology and Biochemistry [41–266]
Postharvest and value adding: 1.755 documents retrieved (Documents H index = 76)	TITLE‐ABS‐KEY ((“*Chenopodium quinoa*” OR “quinua” OR “quinoa” OR “quingua” OR “triguillo” OR “trigo inca” OR “arrocillo” OR “arroz del peru” OR “Kinoa” OR “lagrima de sol”) AND (“nutritional quality” OR “process* method*“OR “protein*“OR “functional*“OR “dry*“OR “phenol*“OR “bioactive compound*“OR “quercetin” OR “kaempferol” OR “flavon*“OR “antioxidant*“OR “saponin” OR “value added”)) AND PUBYEAR >2009 AND (LIMIT‐TO (DOCTYPE, “ar”) OR LIMIT‐TO (DOCTYPE, “re”) OR LIMIT‐TO (DOCTYPE, “cp”)) AND (LIMIT‐TO (SUBJAREA, “AGRI”) OR LIMIT‐TO (SUBJAREA, “CENG”) OR LIMIT‐TO (SUBJAREA, “ENGI”)	Jacobsen S.E.; Københavns Universitet, Department of Plant and Environmental Sciences, Copenhagen, Denmark [39–1.628]	Universidad Nacional Agraria de la Molina, Perú [50–893]	China [282–3.694]	Food Chemistry [82–4.183]
		Arendt, E.K.; APC Microbiome Ireland, Cork, Ireland University College Cork, School of Food and Nutritional Sciences, Cork, Ireland [19–1.641]	Conicet, Argentina [46–673]	United States [145–2.861]	Journal of Cereal Science [42–1.451]
		Murphy, K.M.; Washington State University Pullman, Sustainable Seed Systems Lab, Pullman, United States [17–379]	University of Copenhagen, Denmark [44–1.655]	Italy [127–4.366]	LWT [35–389]
		Zannini, E.; University College Cork, School of Food and Nutritional Sciences, Cork, Ireland [17–820]	Universidad de Chile, Chile [25–1.606]	Spain [123–2.420]	Foods [32–367]
		Repo‐Carrasco‐Valencia, R.; Universidad Nacional Agraria La Molina, Lima, Peru [15–497]	Chinese Academy of Agricultural Sciences, China [41–448]	Argentina [121–2.219]	Journal of Agronomy and Crop Science [30–1.027]
		Vega‐Galvez, A.; Universidad de La Serena, La Serena, Chile [13–833]	Universidad Nacional de Colombia, Colombia [9–55]	Colombia [58–358]	International Journal of Food Science and Technology [27–386]

*Note*: *[Number of documents–number of citations].

### 3.2 Phase 2 and phase 3—Results

This section comprises the obtained results from methodological phases 2 and 3 implementation.

#### 3.2.1 Sowing material and plant breeding knowledge area

**FIGURE 2:**
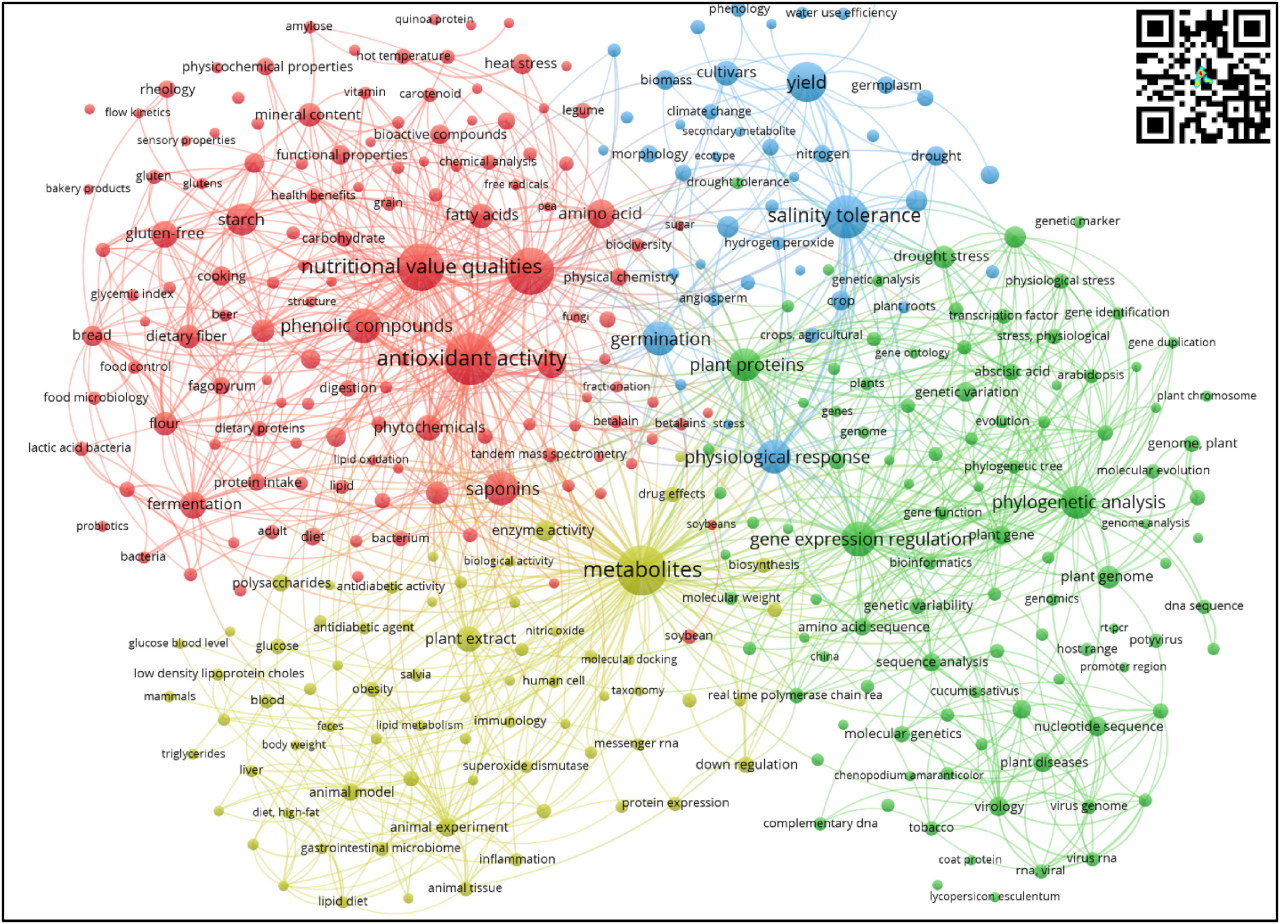
Cooccurrence keyword network—Sowing material and plant breeding. *Source*: Own elaboration based on Scopus®, WoS®, and SciELO® data retrieved in November 2022. Analysis software VOSviewer 1.6.18.

The keyword cooccurrence network analysis technique explained in the Section 2 was implemented with VOSviewer. Figure 2 represents the scientific landscape of quinoa research for the sowing material and plant breeding knowledge area. The network consists of 4 thematic clusters, 342 topics, 8833 connection links, and a total link strength of 15,568 (each topic node has at least two links with other topic nodes).

##### 3.2.1.1 Red cluster (112 key topics)—Bioactive compounds

Plant breeding in quinoa has been focused on variety diversification of nutritional and beneficial properties. Furthermore, enhancing the content of antioxidants, carotenoids, tocopherols, phenols, flavonoids, and starches promotes the functional and nutritional value of quinoa and quinoa‐based foods. According to Graziano et al. (2022), quinoa contains more cysteine, methionine, lysine, flavonoids, phenolic compounds, peptides, starch, fibers, and proteins than cereals such as corn and wheat, positively impacting human health. Therefore, in plant breeding programs for this pseudocereal, the cultivars with the highest amounts of these types of compounds are selected.

##### 3.2.1.2 Green cluster (89 key topics)—genic expression

Research focused on gene expression and regulation includes hybridization (Hodková & Mandák, 2018), phylogenetic (Li et al., 2022), and proteomic studies in relation to edaphoclimatic factors where cultivars are grown (Zhao et al., 2022). The main objective of genic expression studies has been to find the relationship between the agri‐environmental offer and the genetic variability associated with the greater production of seeds with high contents of vegetable proteins (El‐Hakim et al., 2022) and high‐value biocomposites (Campos‐Rodriguez et al., 2022). This is consistent with what was reported by García‐Parra, Stechauner‐Rohringer, et al. (2020) and García‐Parra, Zurita‐Silva, et al. (2020), who indicated that the environmental temperature plays an important role in the phenological phases prior to the development of the seed, influencing productive and compositional aspects. For instance, if the temperature is high in the flowering phase, growth, grain production, and its protein content decrease, so quinoa germplasm must be evaluated under different environmental conditions, and selection must be performed with the ones that best adapt and produce higher yields in each of the evaluated environments (Thiam et al., 2021).

##### 3.2.1.3 Blue cluster (63 key topics)—abiotic stress resistance improvement

Research on plant breeding embraces the generation of adapted cultivars to specific environmental factors such as high drought tolerance (Serna et al., 2020) and salinity soil content (Hussain et al., 2018). This corresponds to what was reported by Shi et al. (2022), who indicate that quinoa has attracted great attention as a crop, mainly because it presents a strong resistance to various adverse climatic and soil conditions, such as cold, high salinity, and drought, which allows quinoa to grow on barren soil. However, abiotic stress can also affect the growth and development of quinoa (Hinojosa et al., 2018). Therefore, recent research on plant breeding has focused on issues related to functional genetics, where the presence of genes in different cultivars is evidenced to better understand the functioning of these genes in the cultivars where they are found and used later in genetic improvement programs (Manjarres‐Hernández et al., 2021).

##### 3.2.1.4 Yellow cluster (62 key topics)—proteins

Research trends indicate a strong relation between quinoa plant breeding programs for increasing protein (Grimberg et al., 2022) and mineral content (Prado et al., 2014), with the promotion of alternative sources of carbohydrates and healthy flours. According to Reguera et al. (2018), the nutritional properties of quinoa seeds are related to the agri‐environmental conditions where they are developed. However, improving the quality and quantity of protein in quinoa seeds is a key challenge for food security. As reported by Gargiulo et al. (2019), proteins are found mainly in the embryo, and the quantity and quality of the protein are directly proportional to the size of the embryo. Therefore, a larger embryo size could be a target trait of interest in quinoa breeding programs for the development of improved varieties with higher protein content.

**FIGURE 3:**
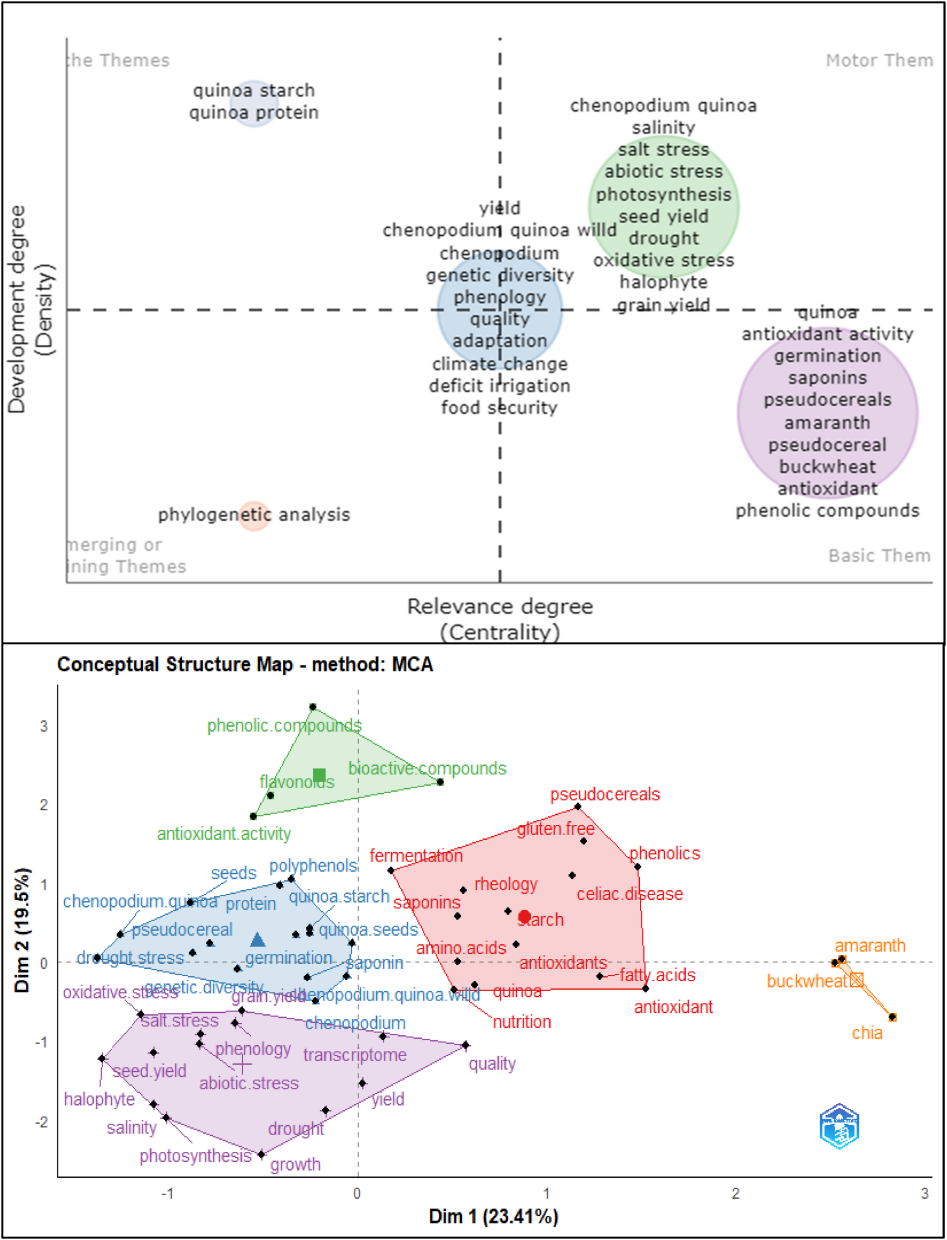
Thematic Map and Factorial Map. *Source*: Own elaboration based on Scopus®, WoS®, and SciELO® data retrieved in November 2022. Analysis software used: Bibliometrix® v 4.1.0.

Figure 3 shows the thematic map for cluster categorization and the factorial map for key research pillar delimitation. The first map synthetizes the research trends, specialization, and diversification. Motor themes (high relevance and development) focus on plant breeding and key genotype identification for drought tolerance, soil salinity, and hydric deficit and their influence on crop yield, physiological efficiency, and photosynthesis function. This is because quinoa adapts to a wide range of degraded soils. Recently, several articles have addressed salt and drought tolerance in quinoa. However, since the quinoa reference genome was published, new transcriptomic studies have been completed on issues associated with tolerance to abiotic stresses. In addition, information on the tolerance of quinoa to other abiotic stress factors, such as frost, UV‐B radiation, and high air temperatures, is limited, which is why research continues in this regard (Afzal et al., 2022). Basic and transversal themes (low development and high relevance) focus on plant breeding to increase phenolic and antioxidant compound contents. The foregoing coincides with what was stated by Hernández‐Ledesma (2019), who indicates that one of the new trends in quinoa research has to do with issues related to chemical constituents and therapeutic properties, which has made this pseudocereal gain recognition as a functional food and nutraceutical, mainly due to the nutritional and biological properties of the seed with emphasis on the bioactive compounds that are mainly responsible for the health benefits attributed to this crop. Niche themes (high development and low relevance) comprise studies on the characterization and improvement of quinoa genotypes with high starch and protein contents. This may be because although there are differences between the genotypes in terms of starch and protein contents, these values do not seem to vary as much (Gargiulo et al., 2019). In addition, it is important to indicate that the amount of these compounds present in the plant is affected by the environment in which they develop (Reguera et al., 2018). Finally, emerging and declining themes (low development and relevance) comprise novel studies on phylogenetic and genetic variability. This occurs because although marker‐assisted selection is much faster than conventional breeding, the need to cross and backcross still has limitations on the progress speed due to the varieties that are used. In addition, the process requires qualified personnel and demands resources, which delays its use in developing countries where investments in research, technology, and training have limited financial resources (Alandia, Rodríguez, et al., 2021).

The second map (factorial representation) shows five pillars for plant breeding research for quinoa crops. The red pillar is focused on varieties and genotypes characterized by their nutritional content enriched with amino acids, saponins, phenolics, fatty acids, and antioxidants. The green pillar represents the top trend of plant breeding focused on bioactive compounds. The blue pillar presents topics related to the improvement of crop yield, germination efficiency, starch and protein content, and drought stress resistance. The purple pillar comprises topics related to phenological and transcriptomic studies, salinity stress resistance, seed yield, and seed abiotic resistance. The small pillar reflects quinoa research on other pseudocereals.

#### 3.2.2 Relations between soil–plant knowledge area

**FIGURE 4:**
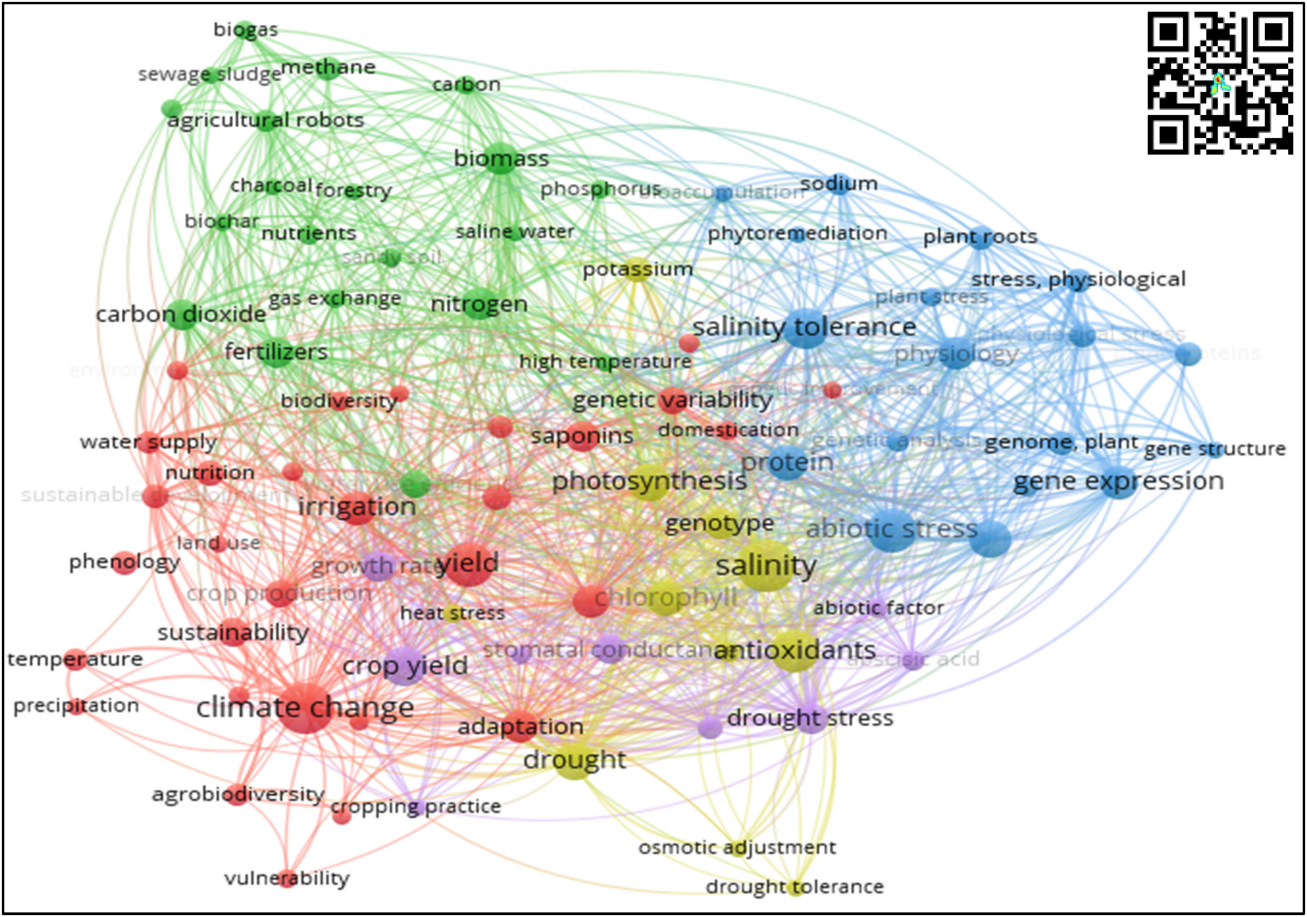
Cooccurrence keyword network—relations between soil–plant scientific landscapes. *Source*: Own elaboration based on Scopus®, WoS®, and SciELO® data retrieved in November 2022. Analysis software VOSviewer 1.6.18.

Figure 4 represents the scientific landscape of quinoa research for the relations between soil–plant knowledge area. The network consists of 4 thematic clusters, 88 topics, 1212 connection links, and a total link strength of 2183 (each topic node has at least two links with other topic nodes).

##### 3.2.2.1 Red cluster (30 key topics)—climate change adaptation

This cluster presents the key research topics on agronomic management focused on adaptability to specific agroecological conditions, conservation practices of soil, and sustainable yield enhancement (Akram et al., 2021; Ruiz et al., 2014; Sellami et al., 2021). Recent studies report the high adaptability of quinoa ecotypes under marginal conditions with extreme levels of temperature, humidity, and soil nutrient availability (AlKhamisi et al., 2021). Furthermore, some studies on genetic improvement focus on the identification of accession conservation status and new breeding programs for climate resilience (Salazar et al., 2019). Finally, research to improve yield under agroclimatic variability, evaluate the influence of different irrigation regimes, and changes in fertilization strategies, including nitrogen inhibitors, biobased inputs, and nanotechnology, is included (Deng et al., 2021; Hasan et al., 2021).

##### 3.2.2.2 Blue and yellow clusters (18 key topics)—physiology and abiotic stress tolerance

This cluster presents research topics on the physiological performance of quinoa varieties in stress environments of salinity, hydric deficit, and drought. Furthermore, research topics comprise the incidence of specific agroecological conditions on biomass, protein, and antioxidant content and bioavailability (Alvar‐Beltrán et al., 2020; Maleki et al., 2019). Salinity stress is a major topic in quinoa crop management research that embraces recent advances in the use of in situ composts for water‐holding capacity improvement and nutritional value increase (El Sebai et al., 2016), although salinity influences quinoa seed antioxidant content mainly due to the presence of heavy metals such as chromium, copper, and cadmium (Guarino et al., 2020). Finally, research on the influence of abiotic stresses on quinoa protein content has focused on the influence of soil amendments (Kakabouki et al., 2014; Sellami et al., 2021) and the use of different seed ecotypes (Veloza Ramirez et al., 2016).

##### 3.2.2.3 Green cluster (20 key topics)—nutrition and fertilization

This cluster comprises specific research topics on soil management and soil nutrition practices with nitrogen and phosphorus for greenhouse gas emissions reduction (Bouras et al., 2022; Pang et al., 2017) and research on biomass production and harvest residue exploitation for biocarbon production as an alternative fertilizer for quinoa cropping systems (Van Minh et al., 2022). The presence of phosphorus and nitrogen by soil bioavailability and fertilization practices influences crop yield as well as the number of grains and panicle peer plants (Jorfi et al., 2022).

##### 3.2.2.4 Violet cluster (10 key topic clusters)

This cluster comprises research focused on crop yield improvement factors such as sowing practices, growth rate indicators, abiotic factors, and drought stress tolerance and resistance (Panuccio et al., 2014; Yasui et al., 2016). Different sowing practices influence the calcium, potassium, magnesium, and protein contents (Zulkadir & İdikut, 2021), especially the use of presowing seed treatments such as glycine betaine to enhance and maintain the growth rate under salinity stress conditions (Maqsood et al., 2021).

**FIGURE 5:**
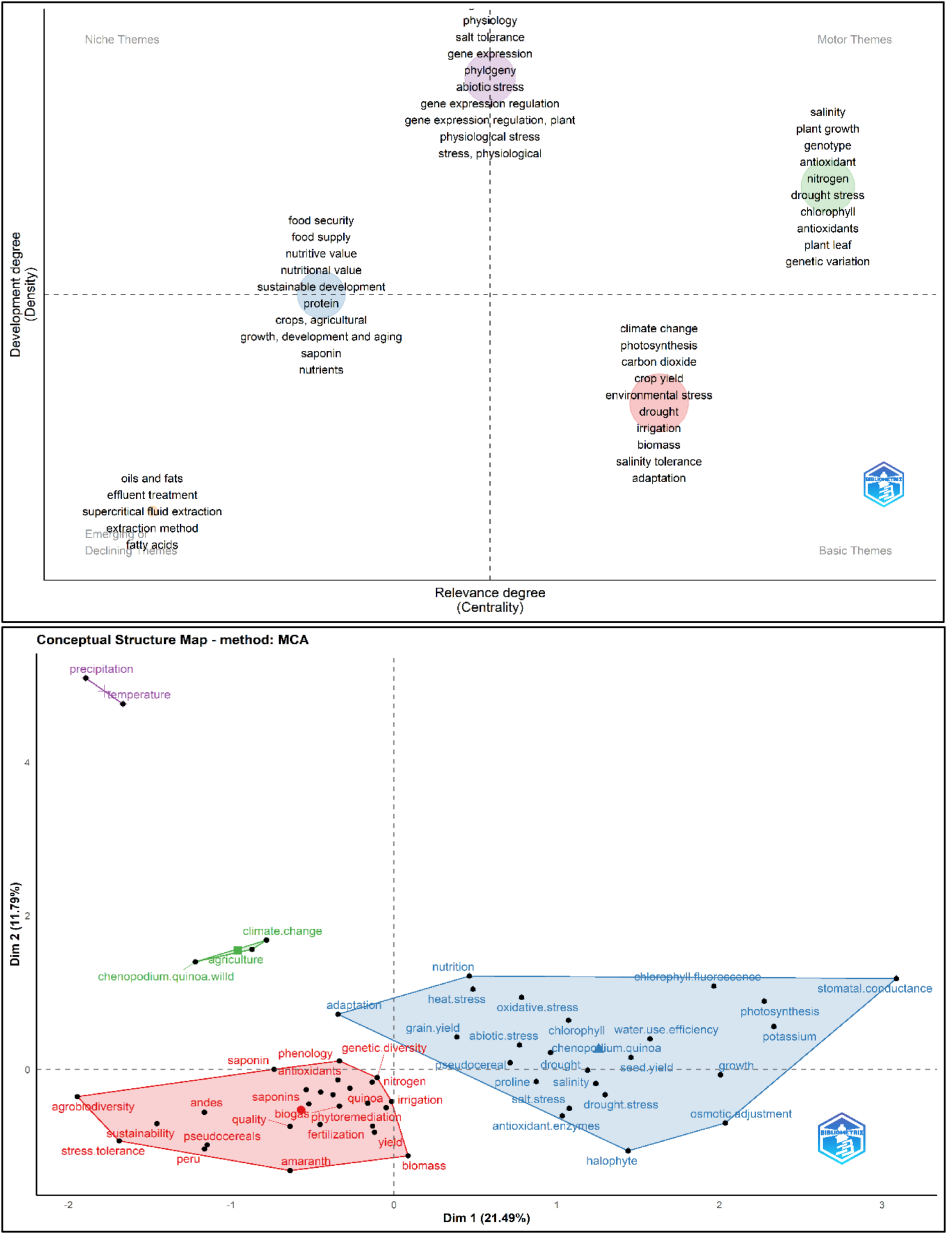
Thematic Map and Factorial Map of Soil–Plant Relations. *Source*: Own elaboration based on Scopus®, WoS®, and SciELO® data retrieved in November 2022. Analysis software used: Bibliometrix® v 4.1.0.

Figure 5 shows the thematic map for cluster categorization and the factorial map for key research pillar delimitation. The first map synthetizes the research trends, specialization, and diversification. Motor themes (high relevance and development) focus on salinity tolerance, plant–soil nitrogen fluxes, and adaptability practices. Emerging and declining themes (low development and relevance) comprise novel studies on agronomic management practices for antioxidant, protein, and fatty acid content improvement. Basic and transversal themes (low development and high relevance) focus on climate change, adaptability, and physiological performance in abiotic stress environments. Finally, niche themes (high development and low relevance) comprise studies on plant breeding of quinoa for abiotic stress resistance.

The second map (factorial representation) shows five pillars for plant breeding research for quinoa crops. The red pillar is focused on studies of crop yield improvement and fertilization programs in salinity, drought, and hydric deficit conditions and their impact on the nutritional content of quinoa. The green pillar represents research focused on balanced crop yield and nutritional value of quinoa mainly in climate change conditions and considering food safety goals. The blue pillar encompasses abiotic stress resistance through plant breeding of varieties with high photosynthesis efficiency. The small pillar includes quinoa abiotic stress and research on climatic factors of temperature and humidity.

#### 3.2.3 Postharvest and value addition activities knowledge area

**FIGURE 6:**
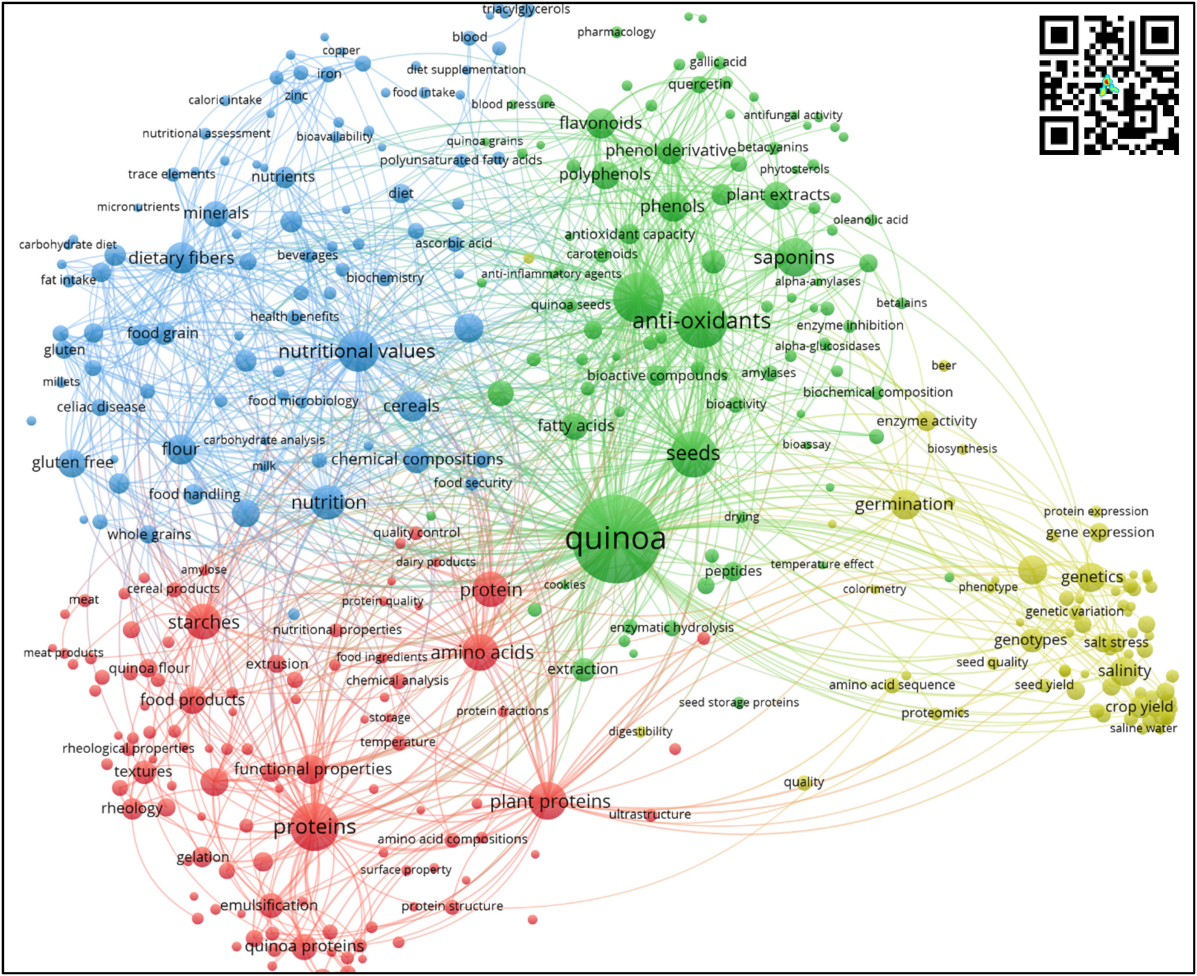
Cooccurrence, Keyword Network—Postharvest and Value‐Adding Scientific Landscape. *Source*: Own elaboration based on Scopus®, WoS®, and SciELO® data retrieved in November 2022. Analysis software used: VOSviewer 1.6.18.

The keyword cooccurrence network analysis technique explained in the Materials and Methods section was implemented with VOSviewer. Figure 6 represents the scientific landscape of quinoa research for the postharvest and value‐adding thematic areas. The network consists of 4 thematic clusters, 380 topics, 13,929 connection links, and a total link strength of 27,297 (each topic node has at least two links with other topic nodes). The co‐occurrence network shows the main topics of recent studies with special attention to the chemical composition of quinoa, for example, “protein,” “nutritional values,” “antioxidants,” and “saponins,” which are variables that depend on genotype, agroecological conditions, and processing.

##### 3.2.3.1 Green cluster—bioactive compounds (97 key topics)

Currently, research on bioactive compounds (phenolic acids, flavonoids, tannins, saponins, polysaccharides, polypeptides, Ecdysone, and aliphatic acids) in quinoa are considered a major trend in food science and food engineering research (Allai et al., 2022), especially for food safety and nutrient uptake improvement due to their high contents of vitamins, proteins, essential amino acids, and minerals (Hu et al., 2018). Furthermore, the extraction of primary and secondary metabolites for nutritional, cosmetic (Bezerra et al., 2021), or pharmaceutical (Valenzuela Zamudio & Segura Campos, 2022) uses, such as polyphenols, flavonoids, anthocyanins, saponins, carotenoids, and tocopherols, embraces the trend of enhancing the antioxidant properties of products (Vidaurre‐Ruiz et al., 2017). In the case of quinoa, the antioxidant function of this pseudocereal is associated with high concentrations of saponins, polyphenols, and other phytochemicals (Tang et al., 2015). The functional properties of these bioactive compounds can enhance other foods with functional properties such as anti‐inflammatory, antihypertensive, antineoplastic, antidiabetic, antimicrobial, antifungal, antiproliferative, and inhibitory activities (Cerdá‐Bernad et al., 2022). Saponins research comprises a specific trend in quinoa bioactive compounds, mainly due to its antibacterial properties against foodborne pathogenic bacteria (Dong et al., 2020); its surfactant properties for bioremediation (Norouzpour et al., 2022); and physicochemical processes for extraction and reduction in saponins content for human consumption such as ultrasound‐assisted extraction (Espinoza et al., 2021), ultra‐pressure extraction (Wang, Wang, et al., 2020; Wang, Zhang, et al., 2020), and response surface methodology (Liang et al., 2017).

##### 3.2.3.2 Blue cluster—food and nutritional safety (97 key topics)

Food nutritional quality has become a worldwide critical gap in food science research, especially in specific nutrient requirements and diets (Saturni et al., 2010). Quinoa is considered a food security crop and a food alternative in the world food basket (Alvarez‐Jubete et al., 2010; Repo‐Carrasco‐Valencia et al., 2010). It is an excellent source of nutritional value, specifically protein, carbohydrate, and essential amino acid contents (Pathan & Siddiqui, 2022). Furthermore, mineral contents of zinc, iron, manganese, calcium, and potassium promote quinoa flour as an important cereal flour (Valdez‐Arana et al., 2020). Another added value of quinoa seed and flour is the dietary fiber content that benefits dairy intake (Craig & Fresán, 2021), cooking properties (Gu et al., 2021), gluten‐free supplementation (Aprodu & Banu, 2021), and antioxidant activities. A major topic for this cluster is the characteristics of quinoa seed and flour nutritional contents of health benefits (i.e., celiac disease), biological availability, and value addition of traditional products (i.e., bakery, pasta, beverages, dairy, and plant‐based protein).

##### 3.2.3.3 Red cluster—plant‐based proteins (111 key topics)

Due to its protein content (both seed and flour), quinoa is considered an alternative source for the design and formulation of plant‐based foods rich in protein (Craig & Fresán, 2021; Jeske et al., 2017). The nutritional, organoleptic, rheological, and physicochemical properties of quinoa proteins have become of great interest as alternative additives in different food formulations due to their emulsification and water absorption, and stability properties (Bansal et al., 2022).

##### 3.2.3.4 Yellow cluster—crop issues related to postharvest activities (75 key topics)

This cluster comprises the interaction between genotypes and the environment (Causin et al., 2020). First, genetic characteristics and the quality of seed material (genotype and phenotype) impact germination, physiological characteristics, and functional expression (Shi & Gu, 2020). Second, soil characteristics such as salinity, water scarcity, and lack of nutrients have a negative impact on crop yield, biomass availability, nutrient content, and bioavailability of phytochemicals (Alasalvar et al., 2021).

**FIGURE 7:**
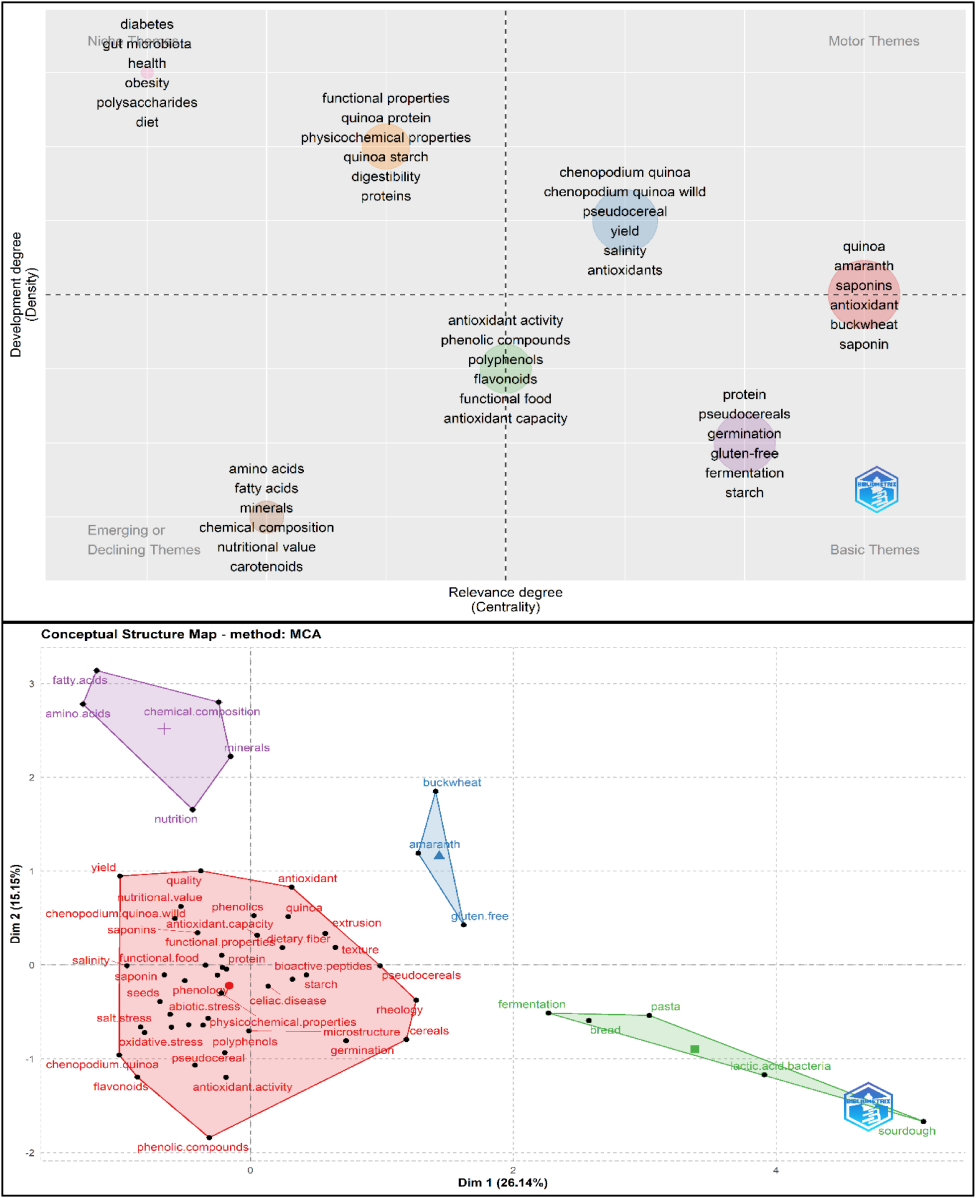
Thematic Map and Factorial Map. *Source*: Own elaboration based on Scopus®, WoS®, and SciELO® data retrieved in November 2022. Analysis software used: Bibliometrix® v 4.1.0.

Figure 7 embraces both the thematic maps and factorial maps for postharvest and added value. The thematic map quadrant classification synthetizes both specialization and diversification clusters.

Motor themes (high relevance and development) focus on quinoa as a pseudocereal with functional properties (antioxidant and saponin content), as well as the incidence of abiotic stresses and bioactive compound content.

Basic themes focus on quinoa not only as an alternative cereal for starch production but also as an alternative source of protein rich in flavonoids and polyphenols in functional food design. Regarding the bioactive properties of quinoa, research has been conducted on hydrolyzed proteins and peptides. Protein‐derived antioxidant peptides have attracted great attention for their effective prevention of oxidative stress and potential application in the food system (Mudgil et al., 2019). Saponins are located in the pericarp of quinoa seeds, which are removed by washing or dry polishing (Suárez‐Estrella et al., 2018). Research has focused on their use in various agricultural applications as a bioinsecticide. Although saponins are considered an antinutritional substance, there are also reports of great interest to the pharmaceutical industry for their antifungal and anticancer properties and cholesterol‐lowering effects (Fuentes & Paredes‐Gonzalez, 2017; Kerwin, 2004).

Niche themes embrace two specialized clusters of health benefits (obesity, diabetes, and gut microbiota) and functional properties of the food industry (assimilable protein content, sensorial characteristics, and digestibility). It has been reported that quinoa protein hydrolysates exhibit antidiabetic effects (Mudgil et al., 2020; Obaroakpo et al., 2019). Additionally, antihypertension (Mudgil et al., 2020), hypolipidemic (Z. Shi et al., 2019), anti‐inflammatory activity (Capraro et al., 2021), and antihemolytic effects have been reported (Mudgil et al., 2019).

Finally, emerging or declining themes focus on the nutritional content of amino acids, minerals, fatty acids, and carotenoids.

Regarding the factorial map, the multicriteria analysis allows a dimensionality reduction in the cooccurrence network and the thematic map (Figure 7). The map represents the four key pillars for current and future quinoa research.

The red pillar includes the added value that confers quinoa as a functional food and as a key component of starch, beverages, plant‐based proteins, pasta, and other bakery products. The violet pillar comprises research on the nutritional and health effects of quinoa consumption. The green pillar comprises research on quinoa transformation processes for bakery products. The blue pillar focuses on research on alternative sources of protein, minerals, and carbohydrates with high added value (i.e., Andean cereals).

### 3.3 Phase 4—Results

**FIGURE 8:**
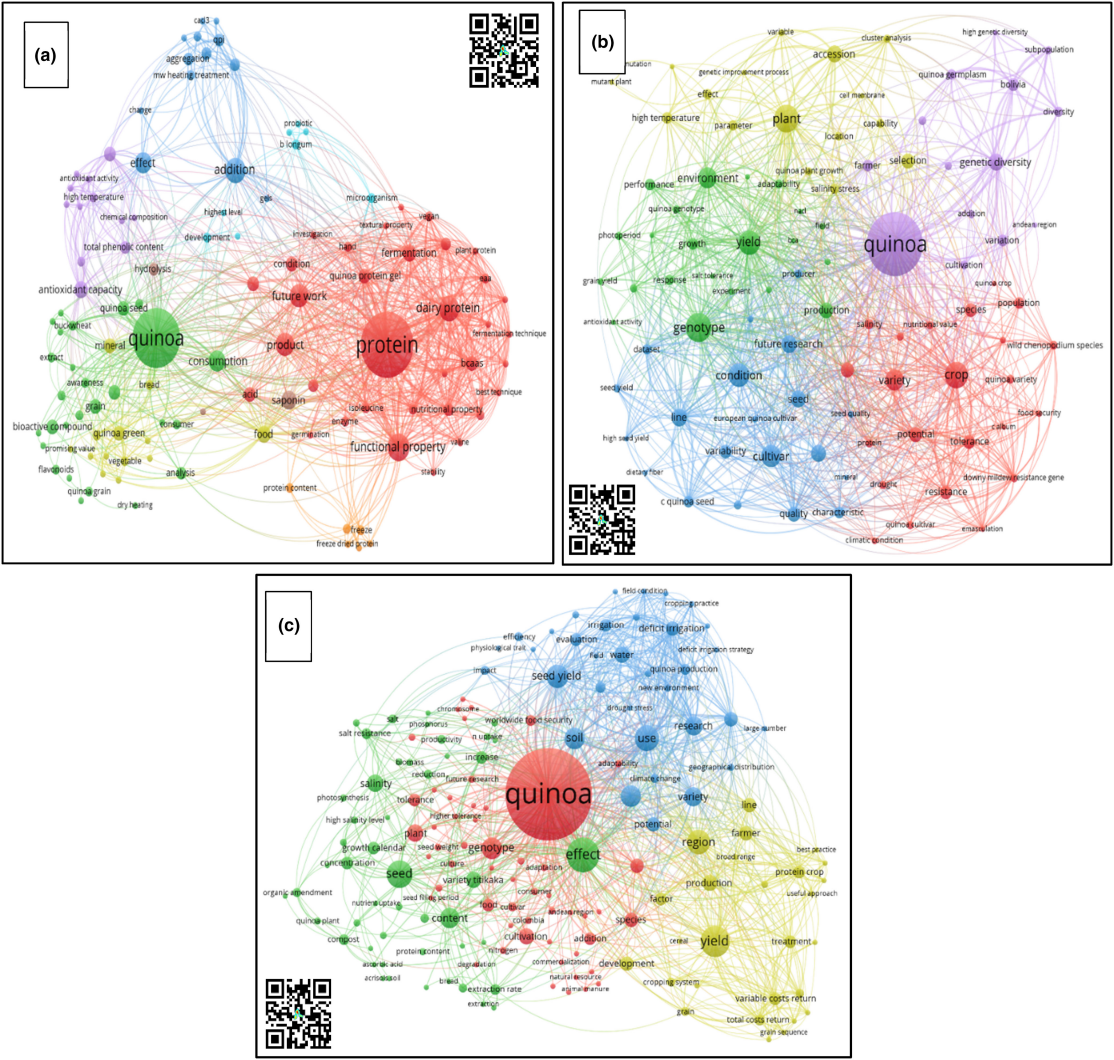
Future Quinoa Research Scientific Landscape. *Source*: Own elaboration based on Scopus®, WoS®, and SciELO® data retrieved in November 2022. Analysis software used: VOSviewer 1.6.18. (a) Postharvest. (b) Soil–plant relations. (c) Plant breeding.

Figure 8 shows the future research perspective of quinoa crops for each thematic area analyzed in the previous sections.

#### 3.3.1 Sowing material and plant breeding thematic area and future scope

As previously mentioned, quinoa is a species that has received great attention, not only in South America but also worldwide, mainly due to its nutritional value and its wide adaptability to different environmental conditions, which is why it is considered one of the most important crops to address food security in the 21st century (Chaudhary et al., 2023). However, as a food and as a crop, quinoa still has a series of limitations, such as increasing yield potential, improving tolerance to some abiotic stress conditions, identifying genotypes that are tolerant to crop‐limiting phytosanitary problems, and generating varieties with low saponin levels in the seeds to obtain sweet genotypes (Opazo‐Navarrete et al., 2018; Suárez‐Estrella et al., 2018), which is highly required in the commercialization of this food. Thus, it could be expected that these are the topics to be addressed in quinoa breeding programs in the short and medium term (Coronado et al., 2022; Manjarres‐Hernández & Morillo‐Coronado, 2022).

Furthermore, quinoa worldwide breeding programs could benefit from 30 years of testing and production outside of the Andean region (120 countries around the globe) that embrace according to the Plant Genetic Resources for Food and Agriculture (PGRFA) global information system 47 countries with gene banks holding accessions (Alandia, Rodríguez, et al., 2021). Regarding traditional breeding (hybridization, interspecific crosses, selection, and mutagenesis), based on gene banks biodiversity, the focus is on key objectives such as yield, selection traits, broad versus narrow adaptation, agronomic characteristics, resistance to abiotic and biotic factors, and end‐use quality (Murphy et al., 2019). Consequently, afford issues, like the identity of the crop, conservation of accessions, mobilization of sustainable utilization, and equity, require scanning and monitoring drivers and barriers related to:

- Genome sequencing for accelerating breeding and genetic enhancement focused on shorter plants with fewer branches, compact seed heads, sweet phenotypes introgression, and reducing saponin content in gene pool (Gao et al., 2021).
- Enhancing adaptability through wild relatives (both diploid and tetraploid) like coastal Chilean quinoa accession (PI 614886), Bolivian Real variety, the *C. berlandieri* ecotype from the northern Gulf of Mexico (var. *boscianum*), and wild *C. hircinum*. These provide insights on promoting nutritional value of quinoa and open up the possibility of targeted breeding of new quinoa varieties (López‐Marqués et al., 2020).
- Exponential and “*game changing*” technologies are critical for addressing the future of food security, sustainability, and climate change, where genetic resources are an essential component of preferable scenarios. Genome editing is a critical issue in agriculture future based on precise gene‐editing tools such as base editing, prime editing, and multiplexing, but, among them, CRISPR/Cas‐based tools have been rapidly repurposed gene edition based on characteristics of improved efficiency, specificity, and reduced off‐target effects. CRISPR/Cas tool provides a robust platform for targeted quinoa breeding (Sharma et al., 2022)
- There is a lack in current legal frameworks for perfectly covering all the issues related to genetic resources and their sustainable management, mainly due to overlaps and discrepancies on geographical levels (local and international), different purposes (genetic resources, varieties and seeds, landscapes, agricultural by‐products, etc.), and inclusion of wild relatives and raw genetic resources of cultivated quinoa, as well as the traditional knowledge related to it (Li, 2022).

Finally, according to Alandia, Odone, et al. (2021), breeding programs must include ethical and intellectual property component embracing the recognition of the origin and sharing practices of genetic materials from the Andean region (seed property rights), which guarantees the equitable distribution of benefits derived from the use of the genetic resources overseas.

#### 3.3.2 Relations between soil–plant thematic area and future scope

One of the strategies to achieve profitability with this crop is to know the suitability of the soil, promoting an improvement in its natural fertility, in addition to ensuring efficient nutritional dynamics through a conventional fertilization plan or with the use of biofertilizers in programs of clean agriculture, which combined with the use of certified seeds, sown at recommended distances and densities, in accordance with the altitude and the genotype to be established, will allow good germination and emergence processes (Garcia‐Parra et al., 2020).

Fertilization is very important in growing quinoa due to its high demand for nutrients. In the field chosen for cultivation, a soil sample must be taken, following the established protocol, to determine the level of available nutrients (Garcia‐Parra et al., 2020; Jacobsen et al., 2003). Depending on the soil, some nutrients required by quinoa may be available in abundance and others in small quantities on the floor; this knowledge will allow you to provide adequate nutrition to achieve high yields and quality. Quinoa responds very well to high fertilization, reaching yields of 6000–7000 kg/ha. In hillside conditions, great amount of soil has low fertility due to the type of soil, the environment, and the continuous planting and harvesting for several years without returning the stolen minerals in each cycle; which is reflected in low yields of around 1000 kg/ha.

Another factor that very strongly influences the availability of nutrients in the soil is the pH. Quinoa thrives very well in a pH range of 5.5–7.8. Consequently, out in these ranges, the availability of nutrients can be strongly affected, causing a reduction in the growth and development of the crop. If they exist acidity problems, or soils with pH <5.5, lime can be applied to slowly increase the pH. Lime can be applied a few months before planting to give lime time to move into the soil profile and change the pH in the root area. On acidic soils, lime should be applied every two or three seasons. depending on the pH change after application and must be incorporated down. If the soil pH is above 7, the soils are alkaline. Floors slightly alkaline may have a low content of manganese (Mn), iron (Fe), zinc (Zn), and boron (B).

Likewise, for the sanitary management of the crop, clean strategies must be included based on the foundations of integrated pest management (IPM), including in strictly necessary cases, the use of chemical synthesis products, minimizing the negative effects on the environment and producer and consumer health. Under these principles, suitable production is expected, but it is the responsibility of the producer to establish a good postharvest plan to satisfy the demand for adequate grain for direct consumption, as well as for the industries' needs. Therefore, this review aims to present some of the technical recommendations that must be considered to achieve the productive and commercial sustainability of quinoa cultivation. Finally, it is advisable to implement deficit irrigation management alternatives as an appropriate strategy to reduce the use of agricultural water and maintain relatively high yields in abiotic stress‐resistant ecotypes (Fghire et al., 2022).

#### 3.3.3 Postharvest and value addition activities, thematic area, and future scope

Quinoa protein is a useful source of bioactive peptides with multifunctional properties, which show benefits for human health. These properties make it a promising ingredient to develop functional foods. Despite the results reported on the functional activity in vitro and in vivo models of quinoa, more research is required. Particularly in studies based on in vivo models with clinical trials to validate the functional properties, as well as unique challenges in discovering its internal mechanisms. The diversification of plant protein use will be essential when animal‐derived proteins fail to satisfy the requirements of the global population in terms of quantity, quality, and added value (Kumar et al., 2022).

## 4 CONCLUSIONS AND FUTURE WORK

Quinoa is an interesting crop appreciated and consumed in different parts of the world for its potential to contribute to food security, which makes it essential to carry out studies that seek to expand the knowledge of this crop.

This research showed that the differences in the yield, content of nutrients, bioactive compounds, and saponins are affected by the interaction of the varietal and the environmental conditions. Thus, the current research topic of quinoa crop breeding is aimed at identifying genotypes that present better gene expression to increase yield in the field according to the environment where they are grown, which is important from agricultural and nutritional perspectives. Additionally, it is necessary to include the topic of stress caused by abiotic factors in the new cultivars and continue with cultivars with a higher content of bioactive compounds and vegetable protein and with low saponin content.

Due to the above and in accordance with current and short‐term trends, it would be expected that to address research issues related to plant breeding in quinoa, as has been done with other species of economic and nutritional interest, the use of both conventional and molecular tools applied to plant breeding to explore and take advantage of the diversity present in the quinoa productive system must be implemented to satisfy the research demands of this crop and incorporate sustainable practices with environmental responsibility for the cultivation of quinoa.

It is important to highlight the relevance of improving varieties adapted to different agroecological conditions, considering the nutritional and functional properties. Moreover, the development of alternative ingredients or raw materials for industrial use to develop new food products or subproducts based on the potential bioactivity of quinoa can be of great value. The information analyzed revealed the importance of carrying out studies that seek to increase the knowledge on the composition of quinoa and the benefits of consuming it, not only for its high nutritional potential but also for its good composition of bioactive compounds. Due to its chemical composition, it can be used as a valuable ingredient in the formulation of foods with value‐added and improved nutritional characteristics with higher contents of all essential amino acids, especially lysine. In the same sense, it is necessary to continue researching the use of saponins with important agricultural pharmacology and cosmetic industrial uses.

## AUTHOR CONTRIBUTIONS

**Diego Hernando Flórez‐Martínez:** Conceptualization (equal); data curation (equal); formal analysis (equal); investigation (equal); methodology (equal); software (equal); validation (equal); visualization (equal); writing – original draft (equal); writing – review and editing (equal). **Jader Rodríguez‐Cortina:** Conceptualization (equal); formal analysis (equal); funding acquisition (equal); investigation (equal); project administration (equal); resources (equal); supervision (equal); validation (equal); writing – original draft (equal); writing – review and editing (equal). **Luis Fernando Chavez‐Oliveros:** Formal analysis (equal); investigation (equal); validation (equal); writing – original draft (equal); writing – review and editing (equal). **Germán Andrés Aguilera‐Arango:** Data curation (equal); formal analysis (equal); investigation (equal); methodology (equal); validation (equal); writing – original draft (equal); writing – review and editing (equal). **Alexis Morales‐Castañeda:** Data curation (equal); formal analysis (equal); investigation (equal); methodology (equal); visualization (equal); writing – original draft (equal); writing – review and editing (equal).

## FUNDING INFORMATION

This study was one of the results derived from the Research Project “Desarrollo de nuevas recomendaciones tecnológicas para contribuir con la competitividad y la sostenibilidad del sector quinuero del departamento del Cauca,” Funded by Colombian “Sistema General de Regalías” from Departamento del Cauca.

## CONFLICT OF INTEREST STATEMENT

The authors declare no conflicts of interest.

## Supporting information


Data S1.

## Data Availability

The data that support the findings of this study are available on request from the corresponding author.
