# Supplementary material for: Current trends and prospects in quinoa research: An approach for strategic knowledge areas
Source: Food Sci Nutr. 2023 Dec 21;12(3):1479–501. doi: 10.1002/fsn3.3891 (PMC10916554; doi:10.1002/fsn3.3891)
Supplement: Supplementary file 1 — Data S1. [file FSN3-12-1479-s001.docx]

**Data S1**

**
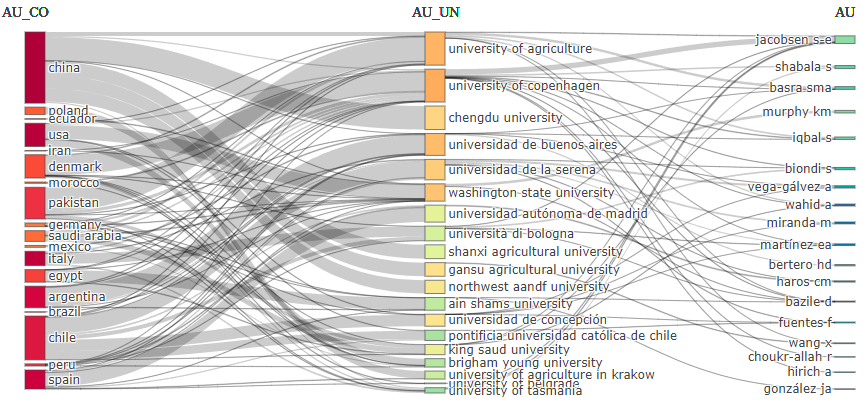
**

**Figure S.1.** Relationships countries, institutions and authors, SankeyPlot - Sowing material and plant breeding.

**Source:** Own elaboration based on Scopus®, WoS® and SciELO® data retrieved in November 2022. Analysis software used: Bibliometrix® v 4.1.0

**
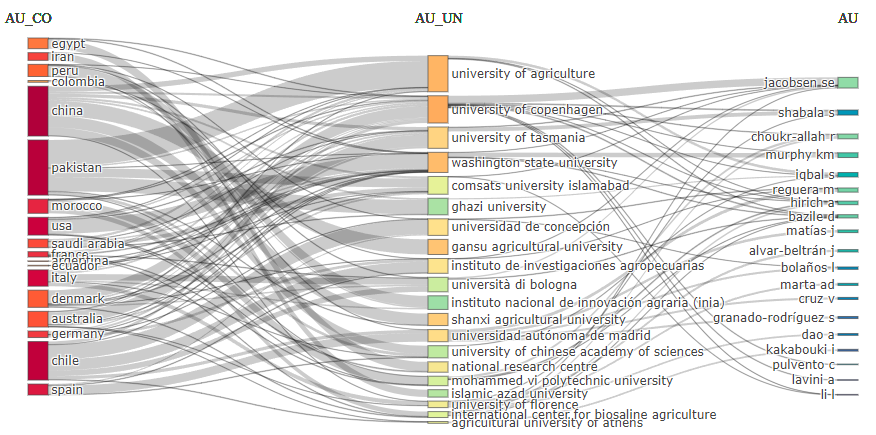
**

**Figure S.2.** Relationships countries, institutions and authors, SankeyPlot - relations between soil‒plant

**Source:** Own elaboration based on Scopus®, WoS® and SciELO® data retrieved in November 2022. Analysis software used: Bibliometrix® v 4.1.0

**
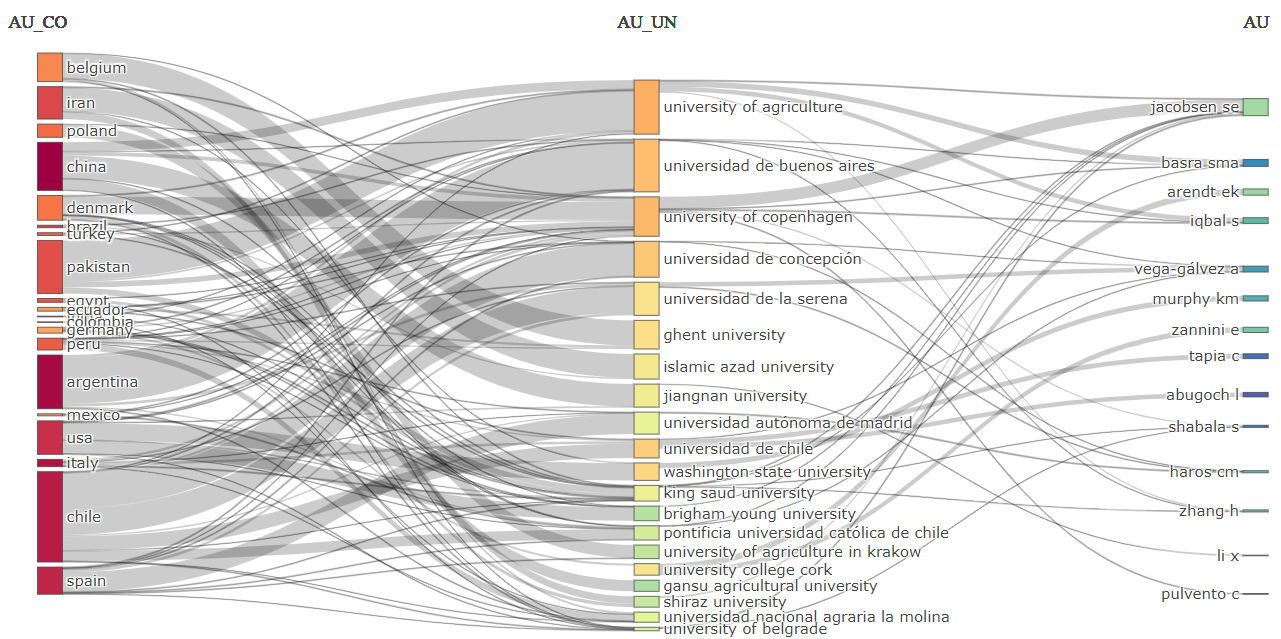
**

**Figure S.3.** Relationships countries, institutions and authors, SankeyPlot - Postharvest and Value-Adding

**Source:** Own elaboration based on Scopus®, WoS® and SciELO® data retrieved in November 2022. Analysis software used: Bibliometrix® v 4.1.0
